# Queen triggerfish *Balistes vetula*: Validation of otolith-based age, growth, and longevity estimates via application of bomb radiocarbon

**DOI:** 10.1371/journal.pone.0262281

**Published:** 2022-01-07

**Authors:** Virginia R. Shervette, Jesús M. Rivera Hernández

**Affiliations:** 1 Fish/Fisheries Conservation Lab, Department of Biology/Geology, University of South Carolina Aiken, Aiken, SC, United States of America; 2 Marine Sciences, SEOE, University of South Carolina, Columbia, SC, United States of America; Ocean Frontier Institute, CANADA

## Abstract

Ensuring the accuracy of age estimation in fisheries science through validation is an essential step in managing species for long-term sustainable harvest. The current study used Δ^14^ C in direct validation of age estimation for queen triggerfish *Balistes vetula* and conclusively documented that triggerfish sagittal otoliths provide more accurate and precise age estimates relative to dorsal spines. Caribbean fish samples (n = 2045) ranged in size from 67–473 mm fork length (FL); 23 fish from waters of the southeastern U.S. (SEUS) Atlantic coast ranged in size from 355–525 mm FL. Otolith-based age estimates from Caribbean fish range from 0–23 y, dorsal spine-based age estimates ranged from 1–14 y. Otolith-based age estimates for fish from the SEUS ranged from 8–40 y. Growth function estimates from otoliths in the current study (L_∞_ = 444, *K* = 0.13, t_0_ = -1.12) differed from spined-derived estimates in the literature. Our work indicates that previously reported maximum ages for *Balistes* species based on spine-derived age estimates may underestimate longevity of these species since queen triggerfish otolith-based ageing extended maximum known age for the species by nearly three-fold (14 y from spines versus 40 y from otoliths). Future research seeking to document age and growth population parameters of *Balistes* species should strongly consider incorporating otolith-based ageing in the research design.

## Introduction

The primary goal of fisheries management is to ensure the long-term sustainable harvest of species while at the same time balancing the cultural, economic, and food security needs of a jurisdiction. This is often achieved through a relatively complex and scientifically rigorous stock assessment process that results in management recommendations. One of the most important inputs for this involves documenting the age structure of a stock [1–3]. Population age structure informs estimates of maturity, mortality, sexual transition for sequential hermaphroditic species, and predictions of lifetime reproductive output [4–7]. Even as jurisdictions move forward with ecosystem-based fisheries management approaches, the documentation of population age structure is still an important underlying component for assessment efforts. Therefore ensuring the accuracy of age estimation through validation is essential when investigating the population demographics of a species [8, 9].

Triggerfishes (Family Balistidae) contribute to productive reef-associated fisheries north and south of the equator in regions of the Atlantic and Indo-Pacific Oceans [10–15]. Queen triggerfish *Balistes vetula* supports local fisheries in jurisdictions throughout the Caribbean Sea and south of the equator in the Atlantic along the coast of Brazil [13, 15–18]. Queen triggerfish is a moderately long-lived species [13, 19] and attains an estimated maximum size of 500 mm FL [20], although fish exceeding 596 mm FL have been reported from port sampling effort in the Caribbean [21]. Queen triggerfish occurs at water depths of up to 275 m and is associated with reef, rubble, and adjacent sandy habitats [20], where it forages for mainly hard-shelled invertebrates such as urchins, crabs, chitons, and bivalves [22, 23]. This species is a nesting benthic spawner and in the north Caribbean forms spawning aggregations during at least some months of its spawning season in associations with the full moon [17, 24]. The annual spawning season for queen triggerfish starts as early as December and extends to August; females in the north Caribbean spawn an average of five times within their protracted spawning season [17].

Bomb radiocarbon is a useful tool in the validation of age estimation for fishes via the application of a region specific Δ^14^C time series [25–28]. Nuclear weapon testing in the 1950s and 1960s resulted in a rapid increase of ^14^C in the atmosphere which subsequently dissolved into oceanic CO_2_ [29–32]. The temporal marine record of the rapid increase and decline in radiocarbon has been documented for multiple oceanic regions across the globe through analysis of Δ^14^C from biogenic carbonate materials from hermatypic corals [30, 33, 34], mollusk shells [32, 35], and fish otoliths [27, 28, 36, 37]. These region specific Δ^14^C time series are used to evaluate fish age estimates through the comparison of fish Δ^14^C measured in otolith core material [25, 28, 38–41] and fish eye lens cores [42–44] that formed during early life. Fish otolith and eye lens cores are metabolically inert tissues once formed and record the isotopic signals from the surrounding waters a fish experienced during the time of tissue formation [45–49]. The Δ^14^C within an otolith core or eye lens core can then be compared to the predicted value from a regional Δ^14^C time series to evaluate the accuracy of age estimates [28, 36, 37, 42]. A Δ^14^C time series was recently developed for waters of the north Caribbean and used successfully to validate age estimation for populations of red hind *Epinephelus guttatus*, mutton snapper *Lutjanus analis*, and white grunt *Haemulon plumieri* [28], yellowtail snapper *Ocyurus chrysurus* [50], and hogfish *Lachnolaimus maximus* [43] from U.S. Caribbean waters.

Triggerfish species are mainly aged using the first dorsal spine, due to the ease of obtaining the spines relative to extracting triggerfish otoliths which are small, fragile, and take more effort to extract [11, 12, 14, 51–53]. However, otoliths are considered to provide more accurate and precise age estimates when compared to alternative structures, like spines, scales, and fin rays, which can significantly underestimate the true age of a fish [54–57]. Past research on queen triggerfish age and growth utilized sections of the first dorsal spine to estimated ages for samples from the north Caribbean (1983–1984) resulting in a maximum estimated age of 7 y [53], and samples from Brazil (1997–1999) resulting in a maximum estimated age of 14 y [19]. However, recent work comparing age estimates in gray triggerfish *Balistes capriscus* from spine sections versus sagittal otoliths indicated that otoliths provide more precise [14] and accurate [58] age estimates. Age estimation validation for queen triggerfish has not been done but is needed to evaluate the accuracy of spine-derived and otolith-derived age estimates for this species.

In waters of the Caribbean, queen triggerfish is considered a data-deficient species due to the lack of information on population demographics. In the U.S. Caribbean, for example, no current information is available on population age structure, sex-specific and combined growth rates, and age-at-sexual maturity despite its importance as one of the top commercially landed reef fish species in U.S. Caribbean waters [15, 17, 59]. The main goal of this study was to determine if sagittal otoliths provide accurate age estimates for a triggerfish species and compare the accuracy and precision of ages obtained from otoliths to those obtained from the first dorsal spine. A secondary goal was to report maximum ages of the species for samples from the northern Caribbean and from Atlantic waters of the southeastern U.S. (SEUS), the northern extent of the species’ geographic range. These results will be utilized to document age, growth, sexual maturity, and mortality for queen triggerfish so that future stock assessments will have these essential data.

## Methods

This study was carried out in strict accordance with the recommendations in the Guide for the Care and Use of Laboratory Animals of the National Institutes of Health. The protocol was approved by the University of South Carolina Aiken Institutional Animal Care and Use Committee (Protocol Number: 053012-BIO-04).

### Fish collection and processing

**Fig 1:**
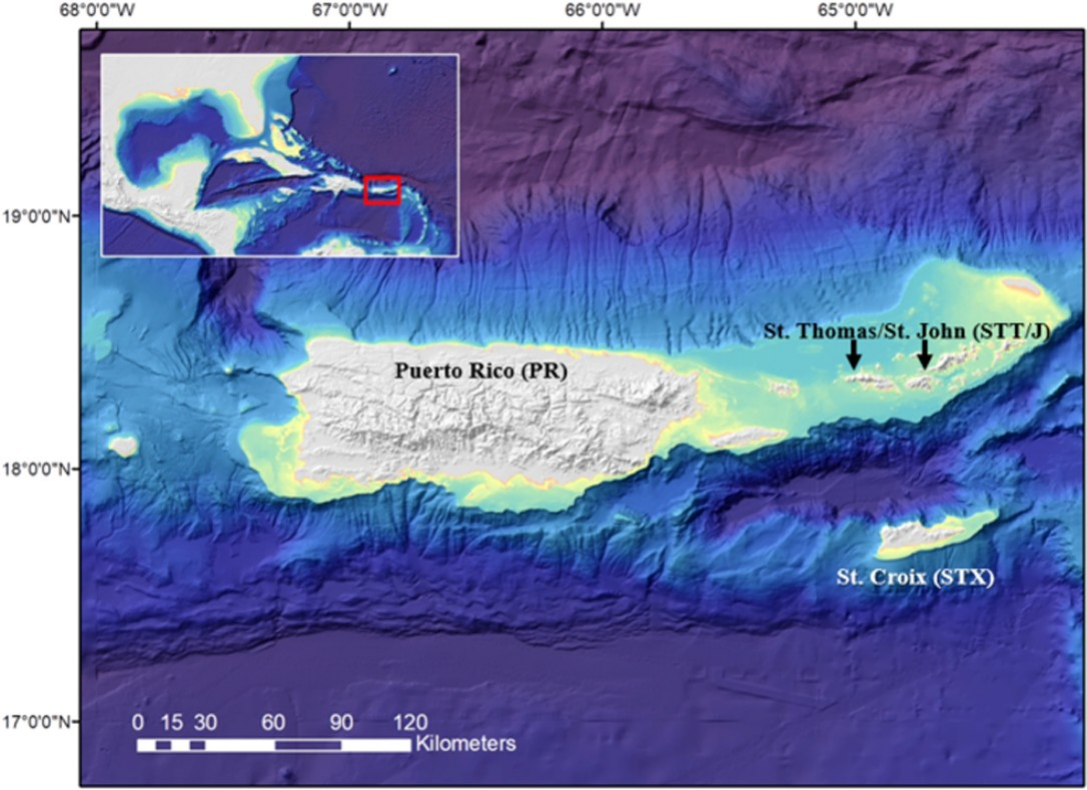
North Caribbean sampling region for queen triggerfish. Map indicates the general north Caribbean region including the major islands of the U.S. Caribbean. The map layer used to generate this figure is from NOAA National Centers for Environmental Information and provided without restriction by the U.S. Government.

Fish samples were collected from waters of the U.S. Caribbean (Fig 1) through fishery-dependent sampling via purchase of fish from local fishers (2013–2021); fishery-independent sampling through the Southeast Area Monitoring and Assessment Program–Caribbean (SEAMAP-C); and opportunistic fishery-independent sampling via collaboration with local fishers (2014–2020). Additionally, we obtained a few large (> 350 mm FL) queen triggerfish from SEUS waters directly from local commercial fishers in South Carolina and North Carolina, U.S. (SC/NC).

All fish were kept on ice until processing occurred. Fish samples were measured for length (SL, FL, TL mm) and whole weight (g). The first dorsal spine was removed, cleaned by removing excess tissue, air-dried, and stored for later processing. Whole eyes were collected from each sample starting in 2018, wrapped in foil and frozen at -21°C. Queen triggerfish otoliths were extracted and stored according to the methods described in Shervette et al. [14]. A detailed protocol is publicly available in http://www.asmfc.org/files/Science/GOM_AtlanticCoast_FishAgeingHandbook_2020web.pdf.

### Age estimation

**Fig 2:**
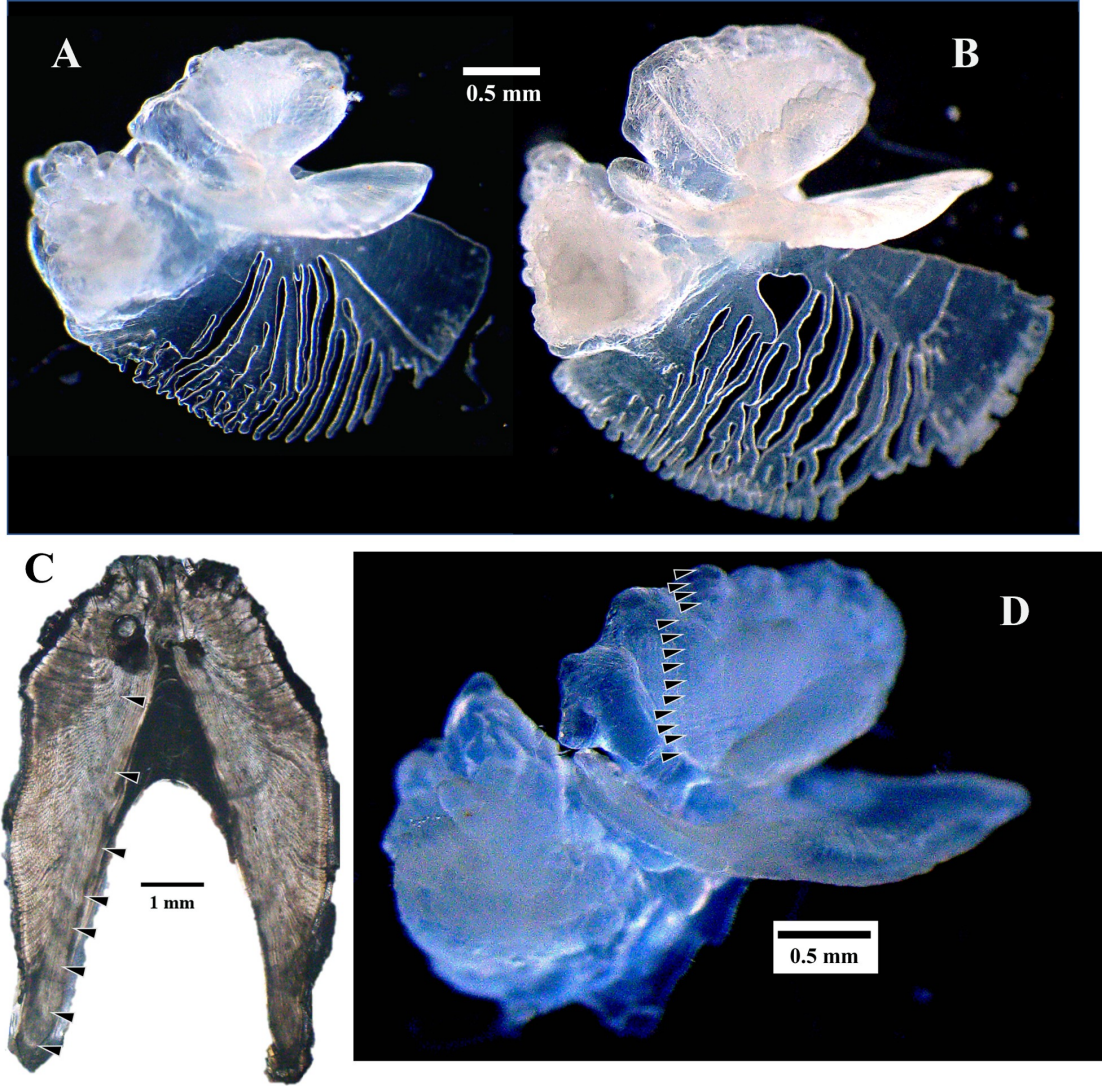
Examples of queen triggerfish sagittal otoliths and comparison between dorsal spine section and sagittal otolith from the same sample. A. Left intact sagittal otolith of a queen triggerfish from a male caught in Puerto Rico (354 mm FL) with 9 increments. B. Left intact sagittal otolith from a male caught in St. Croix (362 mm FL) with 11 increments. C and D. First dorsal spine section (C) and left sagittal otolith (D) with increments indicated on each for a male from St. Thomas (352 mm FL); the spine-based estimated age was 8 y and the otolith estimated age was 14 y.

Sagittal otoliths were read whole for age estimates as described previously in Shervette et al. [14]. Briefly, an otolith was submerged in water and viewed against a black background under a stereomicroscope using reflected light at a magnification of 20-32x. Opaque zones were counted along the sulcular grove region (Fig 2). Sagittal otoliths from each fish were read blind, with no knowledge of fish size, date-of-collection, or sex, by a primary reader (VRS) with over 10 years of experience ageing tropical reef fishes and reading triggerfish otoliths. A subset of otoliths was read independently and blind by a secondary reader (JMRH) with 7 years of experience ageing tropical reef fishes. Average Percent Error (APE) was calculated to assess between-reader precision [60]. Samples for which reader disagreement of opaque zone counts occurred were re-examined simultaneously by both readers and a consensus age estimate was obtained. For each otolith, the location of the last opaque zone was noted relative the otolith edge. The monthly proportion of otoliths with opaque zones on the edge was plotted to determine the annual periodicity of opaque zone formation. This information combined with peak spawning period (January-February; [17]) enabled the establishment of an estimated birthdate (1 February) so that fractional age could be computed for each sample with an otolith age estimate.

The dorsal spine was processed for ageing according to the methods described in Shervette et al. [14] and Kelly-Stormer et al. [52]. A detailed protocol is publicly available in http://www.asmfc.org/files/Science/GOM_AtlanticCoast_FishAgeingHandbook_2020web.pdf. The spine increment count for a sample was determined by examining the dorsal spine section using a stereomicroscope with transmitted light at a magnification of 10-25x. Without knowledge of date-of-collection, fish size, or sex, a reader identified and enumerated the patterns of alternating opaque (faster-growing) and translucent (slower-growing) zones on the dorsal spine section [14]. The total number of increments was equal to the number of translucent zones on a spine (Fig 2). A portion of spine sections was read independently by a second reader and APE was calculated to assess between-reader precision. When a disagreement in increment count occurred for a sample, the two readers examined the spine section together and obtained a consensus increment estimate [52]. Final increment counts (age estimates) from otoliths and dorsal spine sections were compared using an age bias plot [14]. Translucent zone formation for a portion of queen triggerfish from our study was previously determined in Thomas [61] to peak in May. This differed from peak spawning documented from our queen triggerfish samples [17] so fractional ages were not computed for spines.

### Age estimation validation

**Fig 3:**
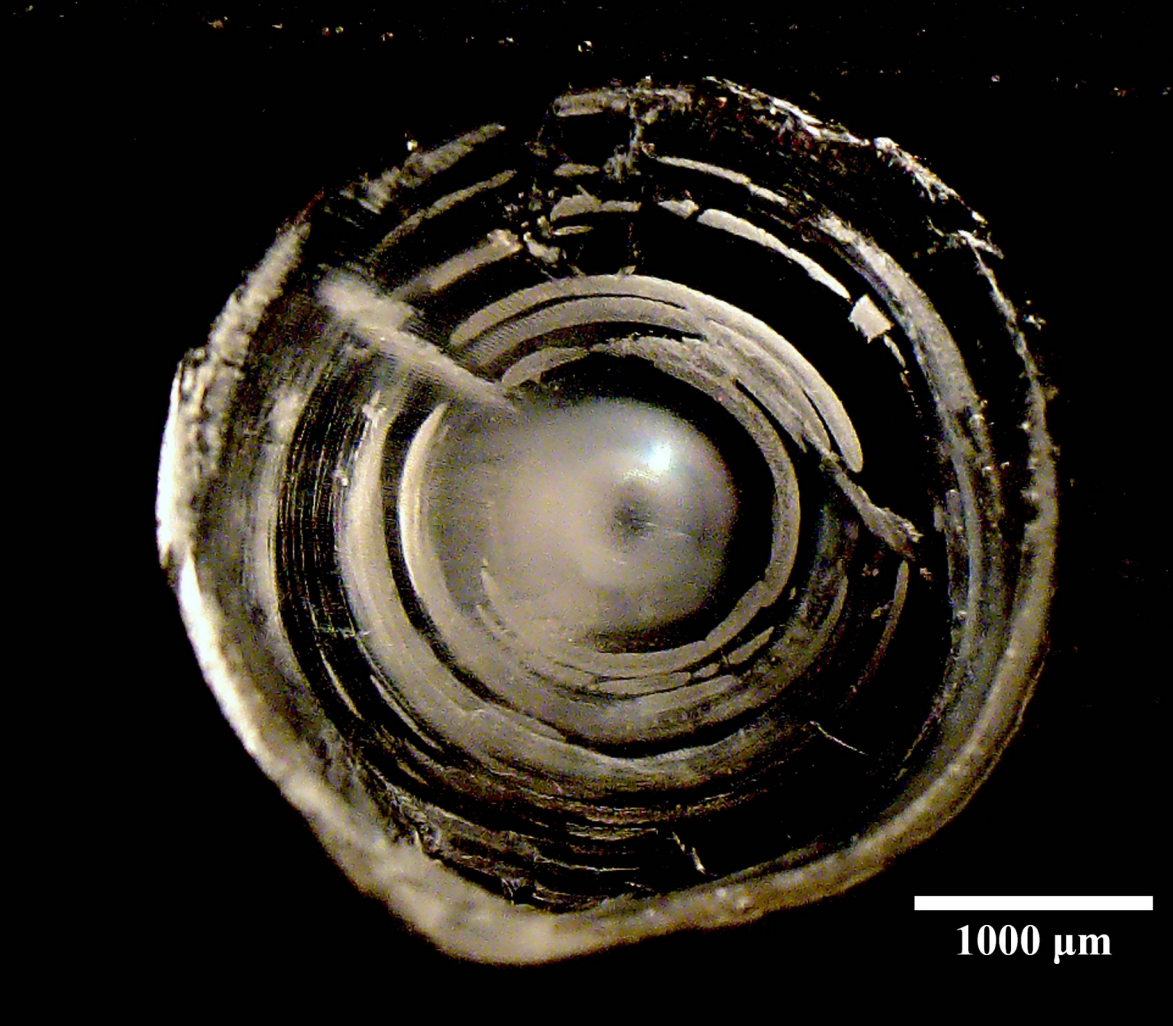
Thin section of a queen triggerfish eye lens. The eye lens core presents as a small pearl-like orb in the center. The diameter of the first major concentric ring after the core was the target size used to obtain eye lens core material representing the first year of life and utilized for Δ^14^C analysis.

Many studies have used otolith cores from fishes for age validation via bomb radiocarbon. However, obtaining Δ^14^C for the birth (hatch) year of a fish via extraction and analysis of otolith core material is not an option for triggerfish otoliths because they are small, fragile, and morphologically inhospitable to precise extraction of core material (Fig 2). Several studies recently demonstrated that eye lens cores of fishes contain archived organic-based chemical signatures from early life [47, 49, 62, 63] and have been successfully used to determine the radiocarbon signature a fish experiences in early life which enabled age estimation [44] and age validation for several marine fishes [43, 50]. Furthermore, a recent study documented that the Δ^14^C signatures of lens and otolith cores were equivalent for several shallow water GOM reef fish species [42]. To establish the target diameter of the eye lens core region that represented the first year of life (hereafter referred to as “core”), we randomly selected 10 queen triggerfish samples, and from the left eyes extracted the whole lens, then allowed the lenses to fully dry. Next, we embedded eye lenses in epoxy resin and once fully hardened, used a low-speed saw with a diamond-edge blade to obtained two thin cross-sections through the center of the lens (~0.5 mm width). Then, we obtained digital images of the lens sections using a camera-stereo microscope system (Fig 3) and measured the diameter of the pearl-like inner core and of the first concentric fracture ring after the inner core for each lens (Fig 3). The mean diameter of the inner core and first concentric ring for the 10 lens samples was 512 μm (32.4 SD) and 1483 μm (103.4 SD), respectively. The first concentric fracture ring mean diameter was similar to the diameter of dried whole eye lenses obtained from the two otolith-aged 1-year old queen triggerfish samples from which we had collected eyes (1510 and 1641 μm). We concluded that a diameter of 1483 μm (~1.5 mm) represented the first year of life for a queen triggerfish and utilized it as the target size for the core regions of the Δ^14^C queen triggerfish validation samples.

A subset of 15 queen triggerfish samples was used to evaluate the accuracy of age estimates from sagittal otoliths and dorsal spine sections via application of the north Caribbean ^14^C time series by measuring the Δ^14^C an individual fish experienced during its first year of life as recorded in the eye lens core region. Forceps and glassware used in the process of obtaining lens cores for Δ^14^C analysis were pretreated to remove any potential carbon contamination by baking in a muffle furnace for a minimum of six hours at a temperature of 500°C. Frozen eye samples were thawed at room temperature and the whole lens was extracted from the left eye of each fish. Lenses were placed in pretreated glass petri dishes and allowed to fully dry. As a lens dries, its concentric outer layers begin to peel back and reveal inner layers. Once a lens was fully dry, the concentric layers were peeled off until the target core region was reached. Each core region was weighed (to the nearest 0.1 mg) and placed in a pretreated glass vial for shipment. Cores were analyzed for Δ^14^C with the accelerator mass spectrometry at the National Ocean Sciences Accelerator Mass Spectrometry facility at Woods Hole Oceanographic Institute (additional information on exact methods used can be found online: www.whoi.edu/nosams/radiocarbon-data-calculations).

Queen triggerfish eye lens core Δ^14^C and corresponding estimated birth year from each ageing structure were overlaid on the north Caribbean Δ^14^C reference time series [28]. The estimated birth year of a sample equaled the year of collection minus the increment count. Peak spawning of queen triggerfish in the north Caribbean occurs January-February [17] so we adjusted the birth year value by adding 0.6 to the original birth year estimate (midpoint in time that core region material formed, assuming fish hatched on 1 February).

Potential ageing bias for each structure was examined by purposely shifting the estimated ages by +/- 1–3 years and superimposing Δ^14^C eye lens core values on the north Caribbean reference Δ^14^C time series. The original age estimates represented an age bias of 0 (null model), while age biases of +1, +2, +3 shifted age estimates to the left (older), and age biases of -1, -2, -3 shifted age estimates to the right (younger). Separately for otolith and dorsal spine-derived age estimates, the sum of squared residuals (SSR) was then computed from predicted versus observed birth years and repeated for the purposely biased age-estimate models [28, 43].

### Growth and size-at-age comparison between ageing structures

For size-at-age data, separate von Bertalanffy growth functions (VBGF) were fit to estimated ages from each ageing structure using the least squares method with the solver function in Microsoft Excel [64]. Calculation of VBGF parameters was repeated with a biologically relevant fixed t_0_ = 0.50 to enable direct comparison of the parameters between the two models. A two-factor ANOVA was used to test the effect of ageing method on estimated size-at-age for ages 5–9, the most prevalent age classes present in the two datasets. The dependent variable for this was FL; the independent variables were age class and ageing structure.

## Results

**Fig 4:**
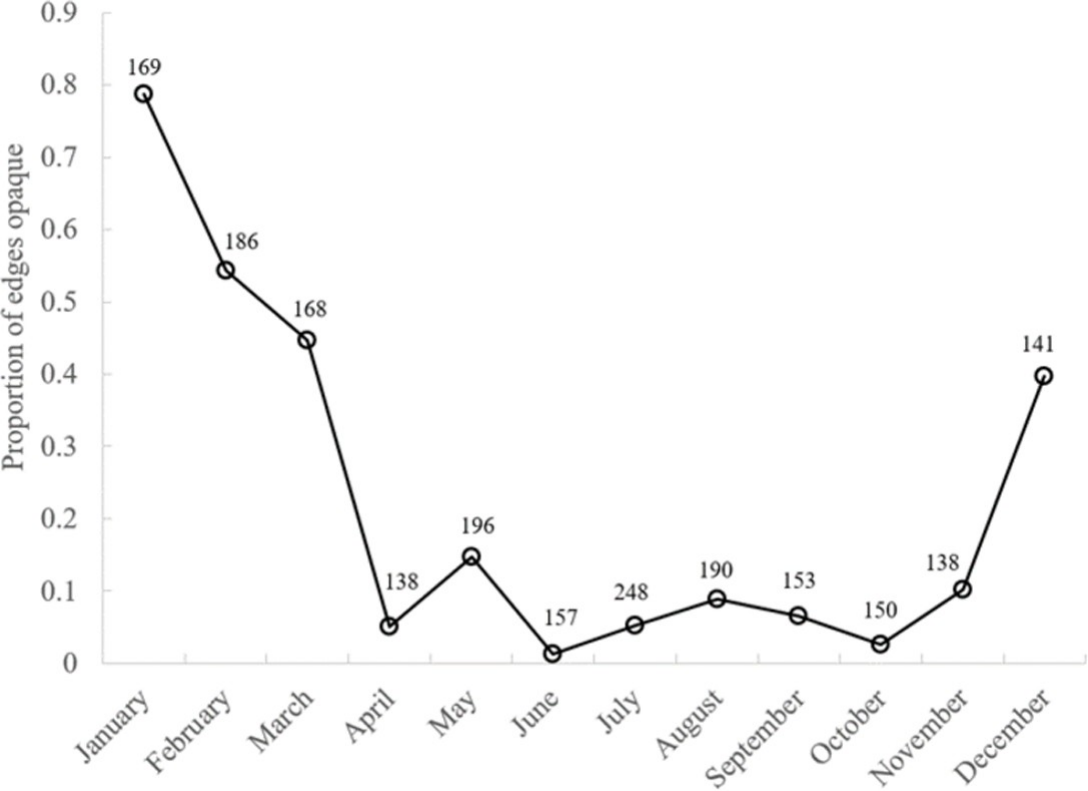
Monthly proportion of otoliths with opaque zones on edges. Number beside each point indicates number of samples per month with otolith edge information.

**Fig 5:**
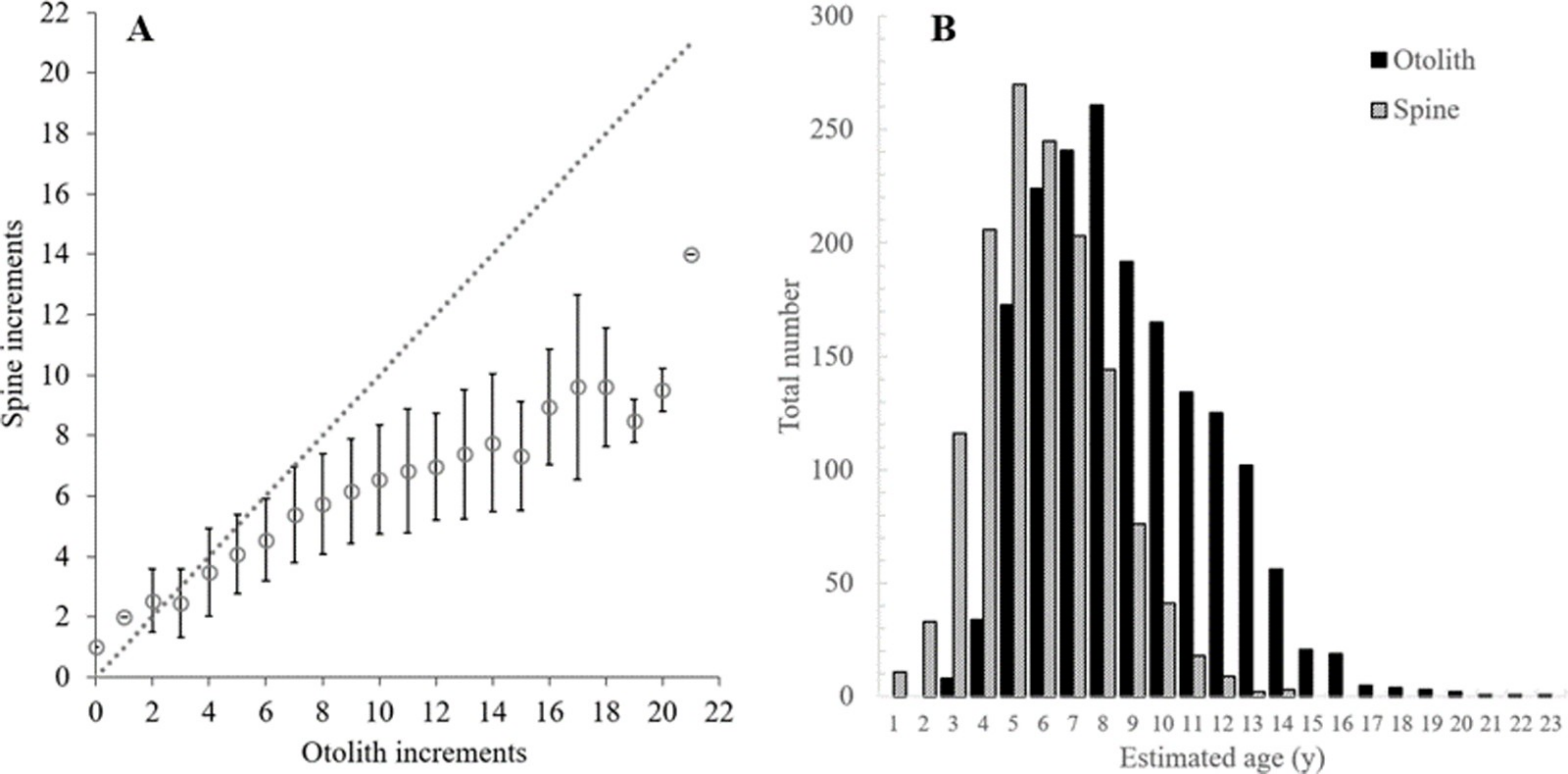
Comparisons between otolith and spine age estimates. A) Age bias plot comparing the age estimates from sagittal otoliths and dorsal spines of individual fish samples; dashed line represents exact agreement of structures for age estimates. B) Comparison of age-class frequency distributions between ageing structures for Caribbean queen triggerfish fishery-dependent samples.

A total of 2164 queen triggerfish was sampled from north Caribbean waters for the overall population demographics study and 23 from NC/SC waters for otolith-based age estimates. Caribbean samples ranged in size from 67–473 mm FL; NC/SC samples ranged from 355–525 mm FL (Table 1). Otolith-based age estimates from Caribbean fish range from 0–23 y, spine-based age estimates ranged from 1–14 y (Table 1). Otolith-based age estimates for NC/SC fish ranged from 8–40 y. A total of 510 and 627 samples had otolith age estimates and spine age estimates, respectively, from the two readers. Between reader APE for otoliths was 3.6% and for spines was 9.0%. Perfect agreement for otolith age estimates occurred for 63% of the samples; 83% had otolith age estimates within 1 y; 96% within 2 y. Perfect agreement for spine age estimates occurred for 52% of the samples; 78% were within 1 y and 90% were within 2 y. An age bias plot indicated that spine age estimates compared to the otolith age estimates underestimated the ages of queen triggerfish starting at age 3 (Fig 5). Opaque zones occurred on otolith edges in relatively high proportion from December-March with a peak in January (Fig 4).

**Table 1:** Queen triggerfish sample summary information.

Category	Time period	N	Size range Mean (mm FL)	Estimated age range Mean (y)
All Caribbean samples	2013–2021	2164	67–473	-
			314	
Samples with otolith age estimates	2013–2021	2045	67–473	0–23
			314	8.4
Samples with spine age estimates	2013–2019	1622	67–473	1–14
			314	5.8
Samples with age estimates from both structures	2013–2019	1587	67–473	-
			314	
NC/SC samples (only aged using otoliths)	2013–2021	23	355–525	8–40
			453	16.4

Time period indicates the years that fish were obtained; N = total number of samples in the category. Summary data were divided into “All Caribbean samples” which includes all of the samples collected to-date for the comprehensive queen triggerfish life history investigation; “Samples with otolith age estimates” includes just the life history samples from the Caribbean with otolith-based age estimates; “Samples with spine age estimates” includes just the life history samples from the Caribbean with spine-based age estimates; “NC/SC samples” includes the samples obtained from southeastern waters of the mainland U.S.A. offshore of North Carolina and South Carolina.

**Fig 6:**
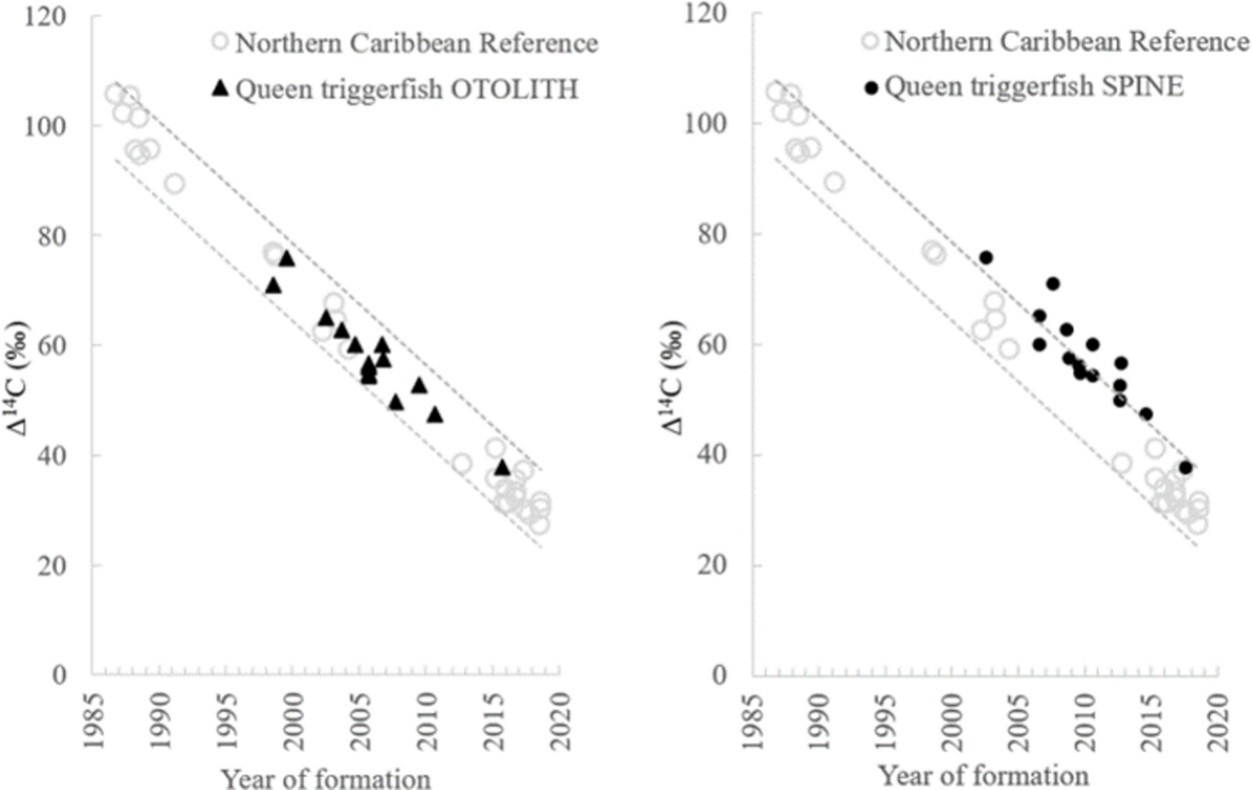
North Caribbean reference Δ^14^C time series with year of formation versus Δ^14^C from otolith-based and spine-based queen triggerfish age estimates. Dashed lines represent upper and lower prediction intervals for the reference Δ^14^C regression relationship.

Bomb radiocarbon (Δ^14^C) results were obtained from eye lens cores for 15 Caribbean queen triggerfish samples ranging in size from 214–473 mm FL. Otolith-based age estimates for the validation samples ranged from 3–21 y with birth year estimates of 1998–2015 (Table 2). All otolith-based ages had birth year estimates versus eye lens core Δ^14^C that fit within the 95% prediction intervals of the north Caribbean reference Δ^14^C time series regression relationship during the ^14^C decline period (Fig 6). Spine-based age estimates from the same 15 samples ranged from 1–14 y with corresponding birth years of 2002–2017 (Table 2). Only 7 of the spine-based age estimates had birth year estimates versus eye lens core Δ^14^C that fit within the 95% prediction intervals of the north Caribbean reference Δ^14^C time series (Fig 6).

**Table 2:** Queen triggerfish eye lens core samples analyzed for Δ^14^C with AMS.

Sample number	Sample year	FL mm	Otolith age	Otolith year of formation	Spine age	Spine year of formation	δ^13^C ‰	Δ^14^C	σ
QUSTT01	2018	214	3	2015	1	2017	-16.70	37.73	2.0
QUSTX01	2018	302	8	2010	4	2014	-16.14	47.5	2.8
QUSTT02	2019	454	12	2007	7	2012	-16.22	49.81	2.1
QUSTT03	2016	285	7	2009	4	2012	-17.49	52.59	2.5
QUSTT04	2019	431	14	2005	9	2010	-16.64	54.39	2.0
QUSTT05	2018	425	13	2005	9	2009	-16.30	54.78	2.1
QUSTX02	2018	392	13	2005	9	2009	-16.77	56.15	2.0
QUSTX03	2018	410	13	2005	6	2012	-15.40	56.68	2.1
QUSTT06	2016	425	10	2006	8	2008	-17.60	57.49	2.2
QUSTX04	2018	410	12	2006	8	2010	-13.16	60.09	2.0
QUSTT07	2019	411	15	2004	13	2006	-16.38	60.11	2.4
QUSTT08	2016	385	13	2003	8	2008	-17.45	62.82	2.3
QUSTT09	2019	438	17	2002	13	2006	-16.76	65.11	2.1
QUSTT10	2019	473	21	1998	12	2007	-14.79	71.01	2.2
QUSTT11	2016	453	17	1999	14	2002	-16.15	75.84	2.2

Year of formation equals sample year minus estimated age.

Results from the ageing bias analysis of eye lens cores Δ^14^C relative to the regression fit of the reference north Caribbean Δ^14^C time series indicated queen triggerfish birth year estimates derived from otolith-based ages were accurate, given that the original otolith-based age estimates had the lowest SSR (129), while the purposefully biased age estimates resulted in SSR values ranging from 139 for +1 y to 1679 for -4 y (Table 3). Ageing bias analysis results for spine-based age estimates indicated that original age estimates were not accurate; the age model with the lowest SSR (182) was the one that utilized an offset of -4 applied to the estimated birth year. This indicates that spine-based age estimates were biased and underestimated fish age by an average of 4 y (Table 3).

**Table 3:** Results from SSR ageing bias analysis.

Age Model	Bias applied years	Otolith SSR	Spine SSR
Null	0	129	1297
-1	-1	278	780
-2	-2	587	422
-3	-3	1054	223
-4	-4	1679	182
+3	+3	632	3800
+2	+2	305	2907
+1	+1	139	1973

Birth year estimates were purposefully biased by -4 to +3 y for otolith-based age estimates and for spine-based age estimates and then the squared residuals from the predicted known-age north Caribbean reference Δ^14^C time series regression were computed.

**Fig 7:**
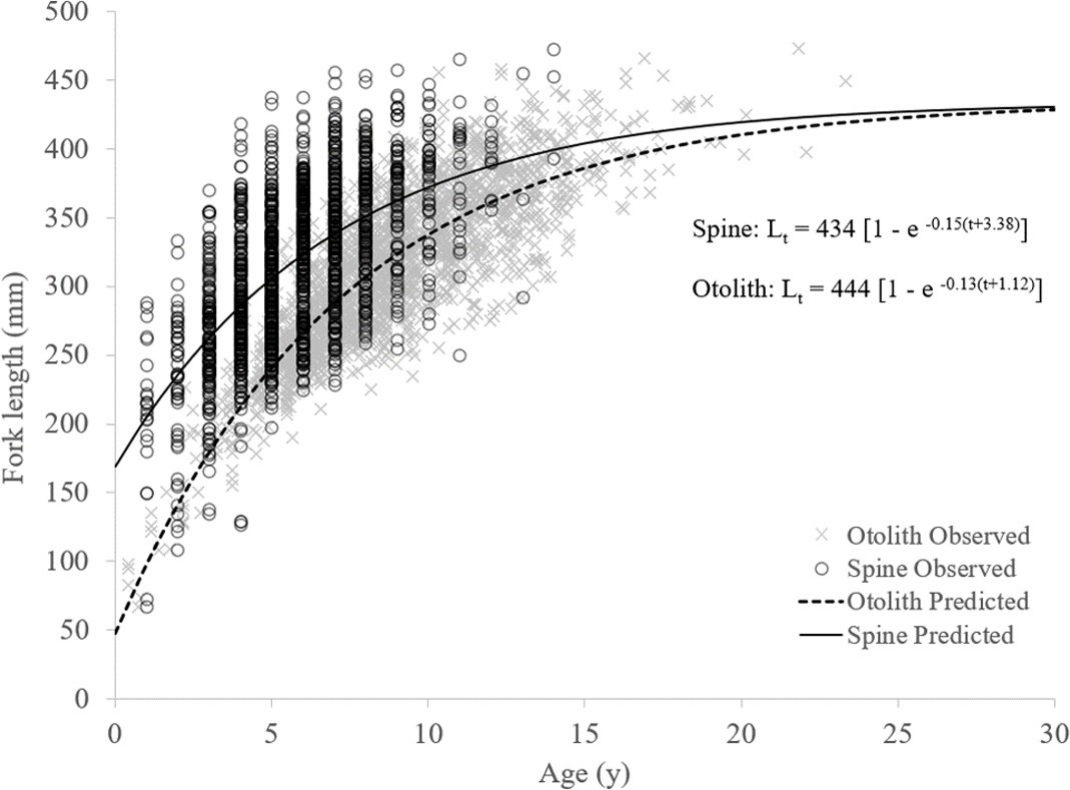
Comparison of length-at-age and von Bertalanffy growth curves based on age estimates from the first dorsal spine versus the sagittal otolith.

Ageing structure-specific growth curves were fitted, yielding the following von Bertalanffy equations: L_t_ = 444 [1—e ^-0.13(t+1.12)^] for otoliths and L_t_ = 434 [1—e ^-0.15(t+3.38)^] for spines (Fig 7 and Table 4). Using a fixed t_0_ = 0.50 resulted in otolith-based ages with L_∞_ = 423 and *K* = 0.15; for spine-based ages, L_∞_ = 368 and *K* = 0.34 (Table 4). The two-factor ANOVA indicated that mean size varied significantly among the age classes (5–9) and between the structures; mean size-at-age was significantly higher for spine-based age estimates across the five age classes (Table 5).

**Table 4:** von Bertalanffy growth function (VBGF) parameters for queen triggerfish.

Model	n	L_∞_	*K*	t_0_	R^2^	P-value
Current study						
Otolith Caribbean	2045	444	0.13	-1.12	0.69	<0.001
Spine Caribbean	1622	434	0.15	-3.38	0.38	<0.001
Otolith t_0_ fixed	2040	423	0.15	-0.50	0.69	<0.001
Spine t_0_ fixed	1622	368	0.34	-0.50	0.35	<0.001
North Caribbean Spine 1980s						
Observed	494	392	0.64	-0.81		
Brazil Spine 1990s						
Observed	476	381	0.34	-0.41		

Parameter estimates from north Caribbean Spine 1980s [52] and Brazil Spine 1990s [19] were included for comparison.

**Table 5:** Results from ANOVA testing for significant differences in mean size-at-age.

Source	df	Sum of Squares	Mean Square	*F*	*P*
Length (FL mm)					
Age (5–9 y)	4	783,947	195,987	122.6	< 0.001
Structure	1	815,036	815,036	509.9	< 0.001
Age x Structure	4	12,233	3058	1.9	0.106
Error	1949	1598	1598		

## Discussion

The current study is the first to directly validate age estimation for queen triggerfish and conclusively document that triggerfish sagittal otoliths provide more accurate and precise age estimates relative to dorsal spines. Our findings support previously published work on gray triggerfish that demonstrated sagittae provided more precise age estimates compared to spines [14] and that otolith-based ages may provide a more accurate representation of population growth parameters. Our work also indicates that previously reported maximum ages for *Balistes* species based on spine-derived age estimates may greatly under-represent longevity of these species since queen triggerfish otolith-based ageing extended maximum known age for the species by nearly three-fold, 14 y from spines versus 40 y from otoliths (Table 5).

### Δ^14^C validation: Otoliths versus spines

Estimated birth years from queen triggerfish otoliths resulted in Δ^14^C values that aligned well with the north Caribbean Δ^14^C reference time series [14]. Most (58%) of the spine-based age estimates for the same samples resulted in birth years that fell above the upper 95% prediction interval limit (Fig 6). The SSR results from the ageing error analysis also indicated that spine-based ages appear to underestimate the true age of fish by around 4 y (Table 3). From the age bias plot (Fig 5) it is clear that spine-based estimates increased in the magnitude of age underestimation with increasing true age of fish. The maximum otolith-based age that was validated in the current study was 21 y, but the spine-based age for the same sample was 14 y (Table 2). Other studies have noted that the agreement between age estimates from otoliths versus external ageing structures progressively worsens with fish age because fewer annuli are discernable on the external ageing structures as age increases [65, 66]. The progressive increase in the magnitude of difference between otolith- and spine-based age estimates means that a simple correction factor applied to spine-based ages reported in past studies for triggerfish species may not be appropriate. The possible development of a more complex conversion to render past spine-based ages for gray triggerfish and queen triggerfish should be explored further.

Various investigators over the past 20 years have observed that, in general, age estimation of fishes “is best achieved” through enumeration of increments that occur in otoliths [8, 9, 67, 68]. The tendency of spines to underestimate ages of triggerfish relative to otoliths may occur because spines accumulate annual increments throughout life, but the interpretation of increments by readers is flawed in that not all increments are detected. However, this seems unlikely when considering the results from several studies that utilized known-age fish to demonstrate that external structures, such as spines, fin rays, and scales, do not continuously accumulate annual increments throughout the life of a fish [55, 69, 70]. Additionally, the first dorsal spine of a triggerfish is utilized as a defensive structure to wedge itself into crevices so that it cannot be easily extracted by predators [52]. This often results in fracturing and breaking of the spine which may impact future increment formation after remodeling of the spine occurs. Future research seeking to document age and growth population parameters of *Balistes* species should strongly consider incorporating otolith-based ageing in the research design.

### Longevity, growth, and population age structure

The use of underestimates for longevity can negatively impact age-based population models [66, 71–73]. Fish longevity is a key input for estimating natural mortality and survivorship [71, 72] and in estimates related to potential lifetime reproductive output [73]. Queen triggerfish age estimation validation results indicated that opaque zone counts on sagittal otoliths provided accurate age estimates, as validated based on application of Δ^14^C in eye lens cores, with the oldest single age directly validated at 21 y. Five additional fish from the sampling regions had ages greater than 21 based on otolith opaque zone counts. Therefore, while 21 was the maximum age for a single otolith sample directly validated with Δ^14^C, the validated ageing method produced a maximum age of 40 y for samples from the current study.

Previous spine-based ageing work on queen triggerfish reported a maximum of 7 y [53] for the north Caribbean and 14 y [19] for the species. Several age estimation validation studies utilizing Δ^14^C published in the past 15 y have noted major increases in longevity estimates for a multitude of fishes [36, 74–77]. One extreme example of this was recently documented in the western Atlantic for warsaw grouper *Hyporthodus nigritus* [77, 78]. Growth increment counts from thin sections of sagittal otoliths of warsaw grouper were validated [77] and the ageing method produced a new maximum age estimate of 91 y for an individual fish [78] that exceeded the previous maximum reported age of 61 by 30 y [76].

The growth parameter estimates obtained from the two ageing structures examined in this study resulted in differences in predicted size-at-age (Table 4 and Fig 7), although when t_0_ was not standardized to a biologically relevant value, *K* and L_∞_ were similar. The spine-based age derived VBGF parameters, when t_0_ = 0.50, yielded a growth coefficient (*K* = 0.34) similar to spine-based age results reported from past queen triggerfish growth models using observed size-at-age (Table 4) [19]. The growth coefficient documented in the current study for otolith-based ages was approximately half of the spine-based age *K* (0.15). A similar difference between growth coefficients from otoliths versus spines was also documented for gray triggerfish [14]. The tendency for spine-based ages to overestimate *K* could lead to problems in evaluating stock status; overestimation of *K* can result in overestimation of stock productivity [79].

Additional complications can result from utilizing age estimates obtained with a method/structure that underestimates true age. For queen triggerfish fishery-dependent samples collected from the north Caribbean, spine-based age estimates resulted in distinct differences in population age structure (Fig 5B). First, the age range was truncated compared to the otolith-based population age structure. Second, the proportion of individuals in younger age classes was higher for spined-based age estimates. These differences in population age structure of fishery-dependent samples between spines and otoliths would lead to differences in estimates of mean age and age at full recruitment to the fishery. The systematic underestimation of age can impede understanding the population dynamics of a species [66, 80] and lead to ineffective management strategies. Our study indicates that spine-based age estimates for queen triggerfish do not result in an accurate understanding of population dynamics, but otoliths do and should be used for triggerfish ageing efforts.

The otolith-based growth estimates reported in this study are preliminary and mainly presented here for comparison with spine-based estimates. A more comprehensive evaluation of regional, gear-related, and sex-related differences in growth for this species will be included as part of a comprehensive publication on queen triggerfish life history. Triggerfish otoliths are small and fragile and these characteristics have precluded past researchers from utilizing them for ageing [52, 81]. However, with proper training on the extraction protocol, they can be easily removed intact. Scientists pursuing the use of otoliths for ageing triggerfishes can adapt the extraction method as needed, once they have some experience in locating otoliths, to reduce external damage to fish samples so that commercial fishers may allow otolith collection more routinely during fishery-dependent port sampling efforts. In the Caribbean, fishers sell their catch whole to consumers and tend to require scientists to purchase fishery-dependent samples for life history research so minimizing damage to a fish is not an issue. However, in places like mainland U.S., state and federal port samplers do not typically purchase fish for life history research and instead they obtain biological samples from fishers through programs that permit them to carefully extract otoliths and gonads from samples that the fishers later sell. We are in the process of developing a second otolith extraction protocol that will minimize external damage to a fish so that port samplers can begin to collect triggerfish otoliths, as well.

Reading whole triggerfish otoliths does require training in handling the otolith and experience in recognizing otolith microstructure patterns to obtain accurate opaque zone counts and ensure high between reader precision. Experience with reading otoliths in general will greatly enhance accuracy and precision, which is typical for ageing work of most subtropical and tropical marine fishes [68]. The two readers in the current study had years of experience with ageing tropical fish species and this is reflected in the low APE and high between-reader agreement. The complexity of ageing fishes is commonly dealt with through the establishment of reference collections and training so should not be an issue for triggerfish species. Our results emphasize that if the research goal of a life history investigation is to obtain accurate and precise age estimates for a triggerfish species, then otoliths should be used.

## References

[1] Ali M, Nicieza A, Wootton RJ. Compensatory growth in fishes: a response to growth depression. Fish and fisheries. 2003;4(2):147–90.

[2] Coleman FC, Figueira WF, Ueland JS, Crowder LB. The impact of United States recreational fisheries on marine fish populations. science. 2004;305(5692):1958–60. doi: 10.1126/science.1100397 15331771

[3] Lorenzen K, Enberg K. Density-dependent growth as a key mechanism in the regulation of fish populations: evidence from among-population comparisons. Proceedings of the Royal Society of London Series B: Biological Sciences. 2002;269(1486):49–54. doi: 10.1098/rspb.2001.1853 11788036 PMC1690856

[4] Collins A, McBride R. Variations in reproductive potential between nearshore and offshore spawning contingents of hogfish in the eastern Gulf of Mexico. Fisheries Management and Ecology. 2015;22(2):113–24.

[5] Gamboa-Salazar KR, Wyanski DM, Bubley WJ, Klibansky N. Effects of age and size on spawning and egg production in gag and scamp grouper off the southeastern United States. ICES Journal of Marine Science. 2020;77(1):290–9.

[6] Pauly D, Morgan G. Length-based methods in fisheries research: WorldFish; 1987.

[7] Ricker WE. Computation and interpretation of biological statistics of fish populations. Bull Fish Res Bd Can. 1975;191:1–382.

[8] Campana SE. Accuracy, precision and quality control in age determination, including a review of the use and abuse of age validation methods. Journal of Fish Biology. 2001;59(2):197–242.

[9] Choat J, Kritzer J, Ackerman J. Ageing in coral reef fishes: do we need to validate the periodicity of increment formation for every species of fish for which we collect age-based demographic data? Tropical fish otoliths: Information for assessment, management and ecology: Springer; 2009. p. 23–54.

[10] Aggrey-Fynn J. The fishery of Balistes capriscus (Balistidae) in Ghana and possible reasons for its collapse: Thesis Doctor of Natural Sciences Faculty 2 (Biology/Chemistry), University …; 2007.

[11] Allman R, Patterson WI, Fioramonti C, Pacicco A. Factors affecting estimates of size at age and growth in grey triggerfish Balistes capriscus from the northern Gulf of Mexico. Journal of fish biology. 2018;92(2):386–98. doi: 10.1111/jfb.13518 29243251

[12] Barroso-Soto I, Castillo-Gallardo E, Quiñonez-Velázquez C, Morán-Angulo RE. Age and growth of the finescale triggerfish, Balistes polylepis (Teleostei: Balistidae), on the coast of Mazatlán, Sinaloa, Mexico. Pacific Science. 2007;61(1):121–7.

[13] Freitas Netto R, Madeira di Beneditto AP. Growth, mortality and exploitation rates of the queen triggerfish Balistes vetula in the Brazilian East coast. Cah Biol Mar. 2010;51:93–9.

[14] Shervette VR, Rivera Hernández JM, Nunoo FKE. Age and growth of grey triggerfish *Balistes capriscus* from trans-Atlantic populations. Journal of Fish Biology. 2021;98(4):1120–36. 10.1111/jfb.14644 33314115

[15] SEDAR. SEDAR 46 Stock Assessment Report Caribbean Data-Limited Species. North Charleston, SC. 2016.

[16] Ferreira de Menezes M. Aspectos da biologia e biometria do Cangulo, Balistes vetula Linnaeus, no nordeste do Brasil. Arq Cien Mar. 1979;19:57–68.

[17] Rivera Hernández JM, Peña Alvarado N, Correa Vélez K, Nemeth R, Appeldoorn R, Shervette V. Queen Triggerfish Reproductive Biology in U.S. Caribbean Waters. Transactions of the American Fisheries Society. 2019;148(1):134–47. doi: 10.1002/tafs.10124

[18] Aiken K. The biology, ecology and bionomics of the triggerfishes, Balistidae. Caribbean coral reef fishery resources Manilla: ICLARM. 1983:191–205.

[19] Albuquerque C, Silva Martins A, de Oliveira Leite Junior N, Neves de Araujo J, Marques Ribeiro A. Age and growth of the queen triggerfish Balistes vetula (Tetraodontiformes, Balistidae) of the central coast of Brazil. Brazilian Journal of Oceanography. 2011;59:231–9.

[20] Robertson DR, Van Tassell J. Fishes: Greater Caribbean Online information system: Smithsonian Tropical Research Institute, Balboa, Panama; 2019. Available from: https://biogeodb.stri.si.edu/caribbean/en/pages.

[21] Stevens MH, Smith SG, Ault JS. Life history demographic parameter synthesis for exploited Florida and Caribbean coral reef fishes. Fish and Fisheries. 2019;20(6):1196–217.

[22] Randall J. Food habits of reef fishes in the West Indies. Stud Trop Oceanogr Univeristy of Miami. 1967;5:655–847.

[23] Reinthal PN, Kensley B, Lewis SM. Dietary Shifts in the Queen Triggerfish, Balistes vetula, in the Absence of its Primary Food Item, Diadema antillarum. Marine Ecology. 1984;5(2):191–5. 10.1111/j.1439-0485.1984.tb00314.x.

[24] Bryan DR, Feeley MW, Nemeth RS, Pollock C, Ault JS. Home range and spawning migration patterns of queen triggerfish Balistes vetula in St. Croix, US Virgin Islands. Marine Ecology Progress Series. 2019;616:123–39.

[25] Campana SE. Use of radiocarbon from nuclear fallout as a dated marker in the otoliths of haddock Melanogrammus aeglefinus. Marine Ecology Progress Series. 1997;150:49–56.

[26] Campana SE. Chemistry and composition of fish otoliths: pathways, mechanisms and applications. Marine Ecology Progress Series. 1999;188:263–97.

[27] Kalish JM. Pre-and post-bomb radiocarbon in fish otoliths. Earth and Planetary Science Letters. 1993;114(4):549–54.

[28] Shervette VR, Overly KE, Rivera Hernández JM. Radiocarbon in otoliths of tropical marine fishes: Reference Δ^14^C chronology for north Caribbean waters. Plos one. 2021;16(5):e0251442. doi: 10.1371/journal.pone.0251442 33979387 PMC8115809

[29] Broecker WS, Peng T-H. Tracers in the Sea. Lamont–Doherty Geological Observatory, Columbia University, Palisades, N.Y.1982. doi: 10.1016/s0022-5347(17)54028-8

[30] Druffel EM, Linick TW. Radiocarbon in annual coral rings of Florida. Geophysical Research Letters. 1978;5(11):913–6.

[31] Toggweiler J, Druffel ER, Key RM, Galbraith ED. Upwelling in the Ocean Basins north of the ACC: 1. On the Upwelling Exposed by the Surface Distribution of Δ^14^C. Journal of Geophysical Research: Oceans. 2019;124(4):2591–608.

[32] Turekian K, Cochran J, Nozaki Y, Thompson I, Jones D. Determination of shell deposition rates of Arctica islandica from the New York Bight using natural 228Ra and 228Th and bomb-produced 14C 1. Limnology and Oceanography. 1982;27(4):737–41.

[33] Druffel EM. Radiocarbon in annual coral rings of Belize and Florida. Radiocarbon. 1980;22(2):363–71.

[34] Knutson DW, Buddemeier RW, Smith SV. Coral chronometers: seasonal growth bands in reef corals. Science. 1972;177(4045):270–2. doi: 10.1126/science.177.4045.270 17815626

[35] Weidman CR, Jones GA. A shell-derived time history of bomb 14C on Georges Bank and its Labrador Sea implications. Journal of Geophysical Research: Oceans. 1993;98(C8):14577–88.

[36] Andrews AH, Barnett BK, Allman RJ, Moyer RP, Trowbridge HD. Great longevity of speckled hind (*Epinephelus drummondhayi*), a deep-water grouper, with novel use of postbomb radiocarbon dating in the Gulf of Mexico. Canadian Journal of Fisheries and Aquatic Sciences. 2013;70(8):1131–40.

[37] Barnett BK, Thornton L, Allman R, Chanton JP, Patterson III WF, Anderson HeE. Linear decline in red snapper (Lutjanus campechanus) otolith Δ^14^C extends the utility of the bomb radiocarbon chronometer for fish age validation in the Northern Gulf of Mexico. ICES Journal of Marine Science. 2018;75(5):1664–71.

[38] Francis RC, Campana SE, Neil HL. Validation of fish ageing methods should involve bias estimation rather than hypothesis testing: a proposed approach for bomb radiocarbon validations. Canadian Journal of Fisheries and Aquatic Sciences. 2010;67(9):1398–408.

[39] Kalish J, Johnston J, Smith D, Morison A, Robertson S. Use of the bomb radiocarbon chronometer for age validation in the blue grenadier Macruronus novaezelandiae. Mar Biol. 1997;128(4):557–63.

[40] Kastelle CR, Kimura DK, Goetz BJ. Bomb radiocarbon age validation of Pacific ocean perch (Sebastes alutus) using new statistical methods. Canadian Journal of Fisheries and Aquatic Sciences. 2008;65(6):1101–12.

[41] Piner K, Wischniowski S. Pacific halibut chronology of bomb radiocarbon in otoliths from 1944 to 1981 and a validation of ageing methods. Journal of Fish Biology. 2004;64(4):1060–71.

[42] Patterson WF, Barnett BK, TinHan TC, Lowerre-Barbieri SK. Eye lens Δ^14^C validates otolith-derived age estimates of Gulf of Mexico reef fishes. Canadian Journal of Fisheries and Aquatic Sciences doi: 10.1139/cjfas-2020-0237 2021.

[43] Shervette VR, Rivera Hernández JM, Drake D, Peña-Alvarado N, Santiago Soler W, Magras J. Caribbean Hogfish: documenting critical life history information for a data-poor species in collaboration with U.S. Caribbean fishers. NOAA COOPERATIVE RESEARCH PROGRAM FINAL REPORT. 2020.

[44] Nielsen J, Hedeholm RB, Heinemeier J, Bushnell PG, Christiansen JS, Olsen J, et al. Eye lens radiocarbon reveals centuries of longevity in the Greenland shark (&lt;em&gt;Somniosus microcephalus&lt;/em&gt;). Science. 2016;353(6300):702. doi: 10.1126/science.aaf1703 27516602

[45] Kalish JM. Otolith microchemistry: validation of the effects of physiology, age and environment on otolith composition. J Exp Mar Biol Ecol. 1989;132(3):151–78.

[46] Secor DH, Henderson-Arzapalo A, Piccoli PM. Can otolith microchemistry chart patterns of migration and habitat utilization in anadromous fishes? J Exp Mar Biol Ecol. 1995;192(1):15–33. 10.1016/0022-0981(95)00054-U

[47] Wallace AA, Hollander DJ, Peebles EB. Stable isotopes in fish eye lenses as potential recorders of trophic and geographic history. PloS ONE. 2014;9(10):e108935. doi: 10.1371/journal.pone.0108935 25279946 PMC4184832

[48] Wride MA. Lens fibre cell differentiation and organelle loss: many paths lead to clarity. Philosophical Transactions of the Royal Society of London B: Biological Sciences. 2011;366(1568):1219–33. doi: 10.1098/rstb.2010.0324 21402582 PMC3061109

[49] Quaeck-Davies K, Bendall VA, MacKenzie KM, Hetherington S, Newton J, Trueman CN. Teleost and elasmobranch eye lenses as a target for life-history stable isotope analyses. PeerJ. 2018;6:e4883. doi: 10.7717/peerj.4883 29888128 PMC5991300

[50] Zajovits SN. Caribbean Yellowtail Snapper *Ocyurus chrysurus*: Filling in Critical Gaps in Life History and Novel Ageing Validation Utilizing Δ^14^C: University of South Carolina; 2021.

[51] Aggrey-Fynn J. Distribution and Growth of Grey Triggerfish, *Balistes capriscus* (Family: Balistidae), in Western Gulf of Guinea. West African Journal of Applied Ecology. 2009;15.

[52] Kelly-Stormer A, Shervette V, Kolmos K, Wyanski D, Smart T, McDonough C, et al. Gray triggerfish reproductive biology, age, and growth off the Atlantic Coast of the Southeastern USA. Transactions of the American Fisheries Society. 2017;146(3):523–38.

[53] Manooch C, Drennon C. Age and growth of yellowtail snapper and queen triggerfish collected from the U.S. Virgin Islands and Puerto Rico. Fisheries Research. 1987;6:53–68.

[54] Buckmeier DL, Irwin ER, Betsill RK, Prentice JA. Validity of otoliths and pectoral spines for estimating ages of channel catfish. North American Journal of Fisheries Management. 2002;22(3):934–42.

[55] Buckmeier DL, Smith NG, Reeves KS. Utility of Alligator Gar Age Estimates from Otoliths, Pectoral Fin Rays, and Scales. Transactions of the American Fisheries Society. 2012;141(6):1510–9. doi: 10.1080/00028487.2012.717520

[56] Buckmeier DL, Snow R, Smith NG, Porter C. Are age estimates for Longnose Gar and Spotted Gar accurate? An evaluation of sagittal otoliths, pectoral fin rays, and branchiostegal rays. Transactions of the American Fisheries Society. 2018;147(4):639–48.

[57] Lozano IE, Llamazares Vegh S, Dománico AA, Espinach Ros A. Comparison of scale and otolith age readings for trahira, *Hoplias malabaricus* (Bloch, 1794), from Paraná River, Argentina. Journal of Applied Ichthyology. 2014;30(1):130–4. doi: 10.1111/jai.12317

[58] Patterson WI, Shervette V, Barnett B, Allman R. Do Sagittal Otoliths Provide More Reliable Age Estimates Than Dorsal Spines for Gray Triggerfish? SEDAR62-WP-17. North Charleston, SC2019.

[59] Matos-Caraballo D. Puerto Rico/NMFS Cooperative Fisheries Statistics Program April 2012 –March 2018 NA07NMF4340039. DRNA Fisheries Research Laboratory, 2018.

[60] Beamish RJ, Fournier DA. A Method for Comparing the Precision of a Set of Age Determinations. Canadian Journal of Fisheries and Aquatic Sciences. 1981;38(8):982–3. doi: 10.1139/f81-132

[61] Thomas S. Age, Growth, and Reproduction of the Queen Triggerfish, *Balistes vetula*, from the U.S. Virgin Islands: University of the Virgin Islands; 2018.

[62] Vecchio JL, Ostroff JL, Peebles EB. Isotopic characterization of lifetime movement by two demersal fishes from the northeastern Gulf of Mexico. Marine Ecology Progress Series. 2021;657:161–72.

[63] Vecchio JL, Peebles EB. Spawning origins and ontogenetic movements for demersal fishes: An approach using eye-lens stable isotopes. Estuarine, Coastal and Shelf Science. 2020;246:107047.

[64] Haddon M. Modelling and quantitative methods in fisheries: CRC press; 2010.

[65] Rocha-Olivares A. Age, growth, mortality, and population characteristics of the Pacific red snapper, *Lutjanus peru*. Fishery Bulletin. 1998;96:562–74.

[66] Yule DL, Stockwell JD, Black JA, Cullis KI, Cholwek GA, Myers JT. How Systematic Age Underestimation Can Impede Understanding of Fish Population Dynamics: Lessons Learned from a Lake Superior Cisco Stock. Transactions of the American Fisheries Society. 2008;137(2):481–95. doi: 10.1577/T07-068.1

[67] Secor DH, Dean JM, Campana SE. Recent developments in fish otolith research. Columbia, SC: University of South Carolina Press; 1995.

[68] Choat JH, Robertson D. Age-based studies on coral reef fishes. In: Sale P, editor. Coral reef fishes: dynamics and diversity in a complex ecosystem. San Diego, CA: Academic Press; 2002. p. 57–80. doi: 10.2527/2002.80102726x

[69] Gu P-h, Xiang J-g, Chen Y-f, Li Y-l, Tang J, Xie S-g, et al. A Comparison of Different Age Estimation Methods for the Northern Snakehead. North American Journal of Fisheries Management. 2013;33(5):994–9. doi: 10.1080/02755947.2013.822445

[70] Klein ZB, Bonvechio TF, Bowen BR, Quist MC. Precision and Accuracy of Age Estimates Obtained from Anal Fin Spines, Dorsal Fin Spines, and Sagittal Otoliths for Known-Age Largemouth Bass. Southeastern Naturalist. 2017;16(2):225–34, 10.

[71] Hewitt DA, Hoenig JM. Comparison of two approaches for estimating natural mortality based on longevity. Fishery Bulletin. 2005;103(2):433.

[72] Hoenig JM. Empirical use of longevity data to estimate mortality rates. Fishery Bulletin. 1983;82(1):898–903.

[73] Secor DH. Longevity and resilience of Chesapeake Bay striped bass. ICES Journal of Marine Science. 2000;57(4):808–15. doi: 10.1006/jmsc.2000.0560

[74] Andrews AH, Burton EJ, Kerr LA, Cailliet GM, Coale KH, Lundstrom CC, et al. Bomb radiocarbon and leadradium disequilibria in otoliths of bocaccio rockfish (Sebastes paucispinis): a determination of age and longevity for a difficult-to-age fish. Marine and Freshwater Research. 2005;56(5):517–28. 10.1071/MF04224.

[75] Andrews AH, Humphreys RLJ, Sampaga JD. Blue marlin (Makaira nigricans) longevity estimates confirmed with bomb radiocarbon dating. Canadian Journal of Fisheries and Aquatic Sciences. 2018;75(1):17–25. doi: 10.1139/cjfas-2017-0031

[76] Barnett BK, Chanton JP, Ahrens R, Thornton L, Patterson III WF. Life history of northern Gulf of Mexico Warsaw grouper Hyporthodus nigritus inferred from otolith radiocarbon analysis. Plos one. 2020;15(1):e0228254. doi: 10.1371/journal.pone.0228254 31978207 PMC6980588

[77] Sanchez PJ, Pinsky JP, Rooker JR. Bomb Radiocarbon Age Validation of Warsaw Grouper and Snowy Grouper. Fisheries. 2019;44(11):524–33.

[78] Sanchez PJ, Rooker JR. Age, growth, and mortality of threatened Warsaw grouper, Hyporthodus nigritus, in the Gulf of Mexico. Fisheries Research. 2021;243:106097. 10.1016/j.fishres.2021.106097.

[79] Gwinn DC, Allen MS, Rogers MW. Evaluation of procedures to reduce bias in fish growth parameter estimates resulting from size-selective sampling. Fisheries Research. 2010;105(2):75–9. 10.1016/j.fishres.2010.03.005.

[80] Mace PM, Fenaughty JM, Coburn RP, Doonan IJ. Growth and productivity of orange roughy (Hoplostethus atlanticus) on the north Chatham Rise. New Zealand Journal of Marine and Freshwater Research. 1990;24(1):105–19. doi: 10.1080/00288330.1990.9516406

[81] Johnson AG, Saloman CH. Age, growth, and mortality of gray triggerfish, *Balistes capriscus*, from the northeastern Gulf of Mexico. Fisheries Bulletin. 1984;82:485–92.

